# 骨髓可溶性B细胞成熟抗原表达在多发性骨髓瘤患者中作为BCMA CAR-T细胞疗效预测指标的研究

**DOI:** 10.3760/cma.j.cn121090-20231020-00220

**Published:** 2024-04

**Authors:** 舒荃 高, 娟 穆, 新 李, 嘉 王, 蕊 崔, 静怡 李, 涛 隋, 琦 邓

**Affiliations:** 南开大学第一中心医院血液科，天津 300192 Department of Hematology, First Central Hospital of Nankai University, Tianjin 300192, China

**Keywords:** 多发性骨髓瘤, 可溶性B细胞成熟抗原, 嵌合抗原受体T细胞, Multiple myeloma, Soluble B cell maturation antigen, Chimeric antigen receptor T cell

## Abstract

**目的:**

探索多发性骨髓瘤（MM）患者骨髓可溶性B细胞成熟抗原（sBCMA）表达对靶向B细胞成熟抗原（BCMA）的嵌合抗原受体T细胞（CAR-T细胞）治疗疗效及安全性的影响。

**方法:**

以2018年1月至2021年12月接受人源化抗BCMA CAR-T细胞临床试验的29例复发/难治MM（RRMM）患者为研究对象。流式细胞术检测抗BCMA CAR-T细胞治疗前后骨髓sBCMA的表达并进行差异比较。

**结果:**

①BCMA CAR-T细胞治疗2个月，20例（68.97％）患者获得总体反应（OR），9例患者仅为病情稳定（SD）或微小缓解（MR）。②20例OR组患者骨髓sBCMA表达治疗前高于治疗后［26 926（18 215, 32 488）ng/L对9 968（6 634, 11 459）ng/L，*P*<0.001］；而MR+SD组患者骨髓sBCMA表达治疗前后差异无统计学意义［41 187（33 816, 47 046）ng/L对33 954（31 569, 36 256）ng/L，*P*＝0.145］；CAR-T细胞治疗前骨髓sBCMA表达OR组低于MR+SD组患者（*P*＝0.005）。③全部29例RRMM患者CAR-T细胞峰值与骨髓sBCMA表达无明显线性相关性（*R*^2^＝0.035，*P*＝0.330）。④sBCMA表达水平与CAR-T细胞治疗不良事件严重程度的相关性：0～1级细胞因子释放综合征（CRS）组（13例）与2～4级CRS组（16例）比较骨髓sBCMA表达差异无统计学意义［32 045（18 742, 40 801）ng/L对29 102（24 679, 38 776）ng/L，*P*＝0.879］；0级免疫效应细胞相关神经毒性综合征（ICANS）组（22例）与1～3级ICANS组（7例）比较骨髓sBCMA表达差异无统计学意义［30 073（19 375, 40 065）ng/L对33 816（22 933, 43 459）ng/L，*P*＝0.763］。

**结论:**

骨髓sBCMA表达与RRMM患者接受BCMA CAR-T细胞治疗的疗效有关，但与不良事件严重程度无显著相关性。或可作为RRMM患者接受BCMA CAR-T细胞治疗的疗效预测标志物。

## Abstract (en)

**Objective:**

The effect of bone marrow soluble B cell maturation antigen（sBCMA）expression on the efficacy and side effects of chimeric antigen receptor（CAR）-modified T-cell-targeting B cell maturation antigen（BCMA）in patients with multiple myeloma（MM）.

**Methods:**

This study involved 29 patients with relapsed or refractory MM（RRMM）who received humanized anti-BCMA CAR-T cell clinical trials from January 2018 to December 2021. The expression of sBCMA in bone marrow before and after anti-BCMA CAR-T cell treatment was detected by flow cytometry and compared.

**Results:**

①Two months after BCMA CAR-T cell treatment, 20 patients（68.97％）achieved an overall response（OR）, whereas nine patients had stable disease（SD）or miner emission（MR）. ②The expression of sBCMA in the bone marrow of 20 patients with OR was higher before treatment than after［26 926（18 215, 32 488）ng/L *vs* 9 968（6 634, 11 459）ng/L; *P*<0.001］; no significant difference was observed in patients with MR and SD［41 187（33 816, 47 046）ng/L *vs.* 33 954（31 569, 36 256）ng/L; *P*＝0.145］; sBCMA expression in patients with OR before CAR-T cell treatment was lower than in patients with MR and SD（*P*＝0.005）. ③No significant linear correlation was found between the peak value of CAR-T cells and sBCMA expression in the bone marrow of all 29 patients with RRMM（*R*^2^＝0.035, *P*＝0.330）. ④No significant difference in sBCMA expression was found between grades 0–1 CRS group（13 patients）and grades 2–4 CRS group［16 patients; 32 045（18 742, 40 801）ng/L *vs* 29 102（24 679, 38 776）ng/L, *P*＝0.879］, nor between grade 0 ICANS group（22 patients）and grade 1–3 ICANS group［seven patients; 30 073（19 375, 40 065）ng/L *vs* 33 816（22 933, 43 459）ng/L, *P*＝0.763］.

**Conclusion:**

sBCMA expression in the bone marrow is related to the efficacy of BCMA CAR-T cell therapy in patients with RRMM, but is not significantly correlated with the severity of adverse events. It may serve as a predictive biomarker for the efficacy of BCMA CAR-T cell therapy in these patients.

尽管自体造血干细胞移植和针对多发性骨髓瘤（MM）的新型药物治疗使MM患者预后明显改善[1]–[4]，但复发/难治MM（RRMM）患者生存期很短[3],[5]。B细胞成熟抗原（BCMA）属于肿瘤坏死家族受体超家族成员的一种跨膜糖蛋白，在几乎所有MM细胞中特异性表达[6]，可作为RRMM患者嵌合抗原受体T细胞（CAR-T细胞）治疗的靶点[7]。抗BCMA CAR-T细胞疗法治疗RRMM的有效率为64％～85％，患者的生存时间显著延长[8]–[10]，但仍有部分患者对BCMA CAR-T细胞治疗反应不佳。一项研究中对149例浆细胞病变患者骨髓可溶性BCMA（sBCMA）表达水平进行分析，发现sBCMA是MM患者预后的重要预测指标[11]。目前骨髓sBCMA的表达水平是否影响RRMM患者BCMA CAR-T细胞的疗效及预后尚不清楚，本研究对29例RRMM患者骨髓sBCMA的表达水平与其对CAR-T细胞治疗的疗效及安全性的影响进行了初步探索。

## 病例与方法

1. 病例资料：本研究为回顾性观察性研究，以2018年1月至2021年12月接受人源化抗BCMA CAR-T细胞临床试验（ChiCTR1800017051、ChiCTR2000033925）的29例RRMM患者为研究对象，治疗后评价疗效未获得总体反应（OR）的患者均接受挽救治疗（包括来那度胺、泊马度胺及髓外病灶的放疗），且均于本研究评价疗效及收集标本之后启动。本研究中CAR-T细胞经我院伦理委员会批准（批件号：2015002X及2020N028KY），所有患者签署知情同意书。诊断符合国际骨髓瘤工作组（IMWG）的诊断标准[12]。收集临床资料包括性别、年龄、骨髓细胞形态学、流式细胞学、免疫固定电泳、染色体等检查结果，以及分期及接受过的治疗线数、预后不良因素等。

2. 诊断和治疗效果评价标准：根据IMWG标准[12]，在抗BCMA CAR-T细胞治疗2个月后评估疗效。临床反应分为严格完全缓解（sCR）、完全缓解（CR）、极好部分缓解（VGPR）、部分缓解（PR）、微小缓解（MR）、病情稳定（SD）和病情进展（PD）。sCR+CR+VGPR+PR为OR。

3. 骨髓sBCMA表达的检测：由北京旷博生物技术股份有限公司采用FACS Canto Ⅱ流式细胞仪（美国BD公司产品）检测sBCMA的表达水平。RRMM患者在抗BCMA CAR-T细胞治疗预处理前、CAR-T治疗2个月进行检测。

4. BCMA CAR-T细胞的制备：本临床试验人源化BCMA CAR-T细胞为第二代（共刺激分子为4-1BB、人源化BCMA CAR慢病毒，上海吉倍公司提供）CAR-T细胞，细胞制备参考我中心既往报道[13]。

5. BCMA CAR-T细胞的输注：预处理方案：氟达拉滨30 mg·m^−2^·d^−1^（−4～−2 d），环磷酰胺300 mg·m^−2^·d^−1^（−4～−3 d），于第0天回输自体人源化抗BCMA CAR-T细胞。平均输注CAR-T细胞（2.09±0.16）×10^6^/kg。

6. 外周血BCMA CAR-T细胞的体内扩增：流式细胞术观察BCMA CAR-T细胞输注后外周血CD3^+^ T细胞中BCMA CAR-T细胞的表达。

7. CAR-T细胞治疗中不良事件观察：在BCMA CAR-T细胞治疗中观察不良事件，包括生命体征、血氧、临床全身症状监测，血细胞学及肝脏、肾脏、心脏功能监测，评价安全性。不良反应重点关注细胞因子释放综合征（CRS）和免疫效应细胞相关神经毒性综合征（ICANS），按照美国移植与细胞治疗学会（ASTCT）共识进行分级[14]。

8. 统计学处理：使用SPSS 27.0软件进行数据分析，连续变量采用秩和检验，以中位数（P_25_，P_75_）表示；分类变量采用卡方检验，以例数（百分比）表示；相关性比较采用Pearson相关性分析，*P*<0.05为差异有统计学意义。

## 结果

一、一般资料

29例RRMM患者中，男15例，女14例，中位年龄62（37～78）岁。患者基线一般资料见表1。

**表1:** 29例接受BCMA CAR-T细胞治疗RRMM患者基线一般资料

项目	数值
年龄[岁，*M*（范围）]	62（37~78）
性别[例（%）]	
男	15（51.72）
女	14（18.27）
骨髓MM细胞比例（%，*x±s*）	41.31±15.82
M蛋白（g/L，*x±s*）	27.79±13.32
ISS分期[例（%）]	
1期	0（0）
2期	8（27.59）
3期	21（72.41）
R-ISS分期[例（%）]	
1期	0（0）
2期	20（68.97）
3期	9（31.03）
细胞遗传学危险分层[例（%）]	
低危	15（51.72）
中危	6（20.69）
高危	8（27.59）
合并髓外病灶[例（%）]	11（37.93）
ECOG评分[例（%）]	
0分	9（31.03）
1分	14（48.27）
2分	6（20.69）
3分	0（0）
接受治疗线数（*x±s*）	4.76±1.16
既往接受自体造血干细胞移植[例（%）]	7（24.14）

**注** CAR-T细胞：嵌合抗原受体T细胞；BCMA：B细胞成熟抗原；RRMM：复发难治性多发性骨髓瘤；ISS：国际骨髓瘤分期；R-ISS：修正的国际骨髓瘤分期；ECOG评分：美国东部肿瘤协作组体能状况评分

二、BCMA CAR-T细胞治疗的疗效及安全性

CAR-T细胞治疗后2个月进行疗效评价，20例（68.97％）患者获得OR，其中10例达sCR/CR，4例达VGPR，6例达PR；其余9例患者为MR或SD。CAR-T细胞输注后检测体内扩增情况，峰值出现在细胞输注后第7～14天，14 d后逐渐下降。OR组患者CAR-T细胞峰值为（33.23±17.45）％，高于MR+SD组患者的峰值［（4.92±2.25）％，*t*＝7.125，*P*<0.001］。

CAR-T细胞治疗过程中，28例患者发生CRS，其中1级12例（41.38％）、2级13例（44.83％）、3级1例（3.45％）、4级2例（6.90％）；7例患者发生ICANS，其中1级4例（13.79％）、2级2例（6.9％）、3级1例（3.45％），无患者发生4级ICANS。其他不良事件以发热或伴寒战、头痛、心动过速、恶心、咳嗽、水肿等临床表现为主。

三、骨髓sBCMA表达水平

1. sBCMA表达水平：29例RRMM患者在BCMA CAR-T细胞治疗前骨髓sBCMA表达水平为30 731（19 441, 40 801）ng/L；以同期我院非CAR-T细胞治疗初治获得OR的16例MM患者为对照组，治疗前sBCMA表达水平为25 781（21 291,36 292）ng/L，差异无统计学意义（*P*＝0.538）。RRMM患者在BCMA CAR-T细胞治疗后骨髓sBCMA表达水平为11 231（6 972, 26 875）ng/L，而对照组治疗后骨髓sBCMA表达水平为22 596（14 944, 28 947）ng/L，差异无统计学意义（*P*＝0.072）。

2. RRMM患者是否伴有髓外病灶sBCMA表达：在CAR-T细胞治疗前，11例伴髓外病灶的RRMM患者骨髓sBCMA表达水平为37 856（26 425, 45 405）ng/L，18例不伴有髓外病灶患者表达水平为29 706（17 161, 33 816）ng/L，差异无统计学意义（*P*＝0.087）。

3. 接受CAR-T细胞治疗获得不同疗效患者治疗前后sBCMA表达：OR组患者骨髓sBCMA表达，治疗前为26 926（18 215, 32 488）ng/L，治疗后为9 968（6 634, 11 459）ng/L，差异具有统计学意义（*P*<0.001）。MR+SD组患者骨髓sBCMA表达，治疗前为41 187（33 816, 47 046）ng/L，治疗后为33 954（31 569, 36 256）ng/L，差异无统计学意义（*P*＝0.145）。治疗前骨髓sBCMA表达在OR组患者低于MR+SD组患者，差异有统计学意义（*P*＝0.005）。

4. sBCMA表达水平与CAR-T细胞峰值的相关性：29例RRMM患者CAR-T细胞峰值与骨髓sBCMA表达水平无明显线性相关性（*R*^2^＝0.035，*P*＝0.330）。

5. sBCMA表达水平与CAR-T治疗不良事件严重程度的相关性：全部29例接受CAR-T细胞治疗的RRMM患者，0～1级CRS组13例骨髓sBCMA表达水平为32 045（18 742, 40 801）ng/L，与2～4级CRS组16例的29 102（24 679, 38 776）ng/L比较，差异无统计学意义（*P*＝0.879）；22例0级ICANS组骨髓sBCMA表达水平为30 073（19 375, 40 065）ng/L，与7例1～3级ICANS组的33 816（22 933, 43 459）ng/L比较，差异无统计学意义（*P*＝0.763）。

6. 骨髓瘤细胞BCMA表达水平与疗效及不良事件严重程度的相关性：29例RRMM患者CAR-T治疗前骨髓瘤细胞BCMA表达水平为（58.23±32.60）％，与CAR-T细胞疗效分层（*R*^2^＝0.007，*P*＝0.655）、CRS分级（*R*^2^＝0.083，*P*＝0.129）、ICANS分级（*R*^2^＝0.174，*P*＝0.024）均无明显线性相关性。

## 讨论

针对RRMM患者靶向BCMA的CAR-T细胞和基于抗体的疗法疗效显著[15]，BCMA CAR-T细胞治疗有望成为长期控制疾病的有效治疗手段[8]–[10]。CAR-T细胞治疗肿瘤根除不彻底及后续疾病再进展等问题有待解决[16]，其中一个重要的原因是靶细胞抗原BCMA通常在MM细胞中低水平表达，MM细胞BCMA抗原表达的异质性限制了该疗法的疗效[8],[17]。

BCMA被普遍存在的多亚基γ-分泌酶复合物从浆细胞表面切割，释放sBCMA片段[18]，sBCMA导致肿瘤细胞上配体密度降低，抑制BCMA CAR-T细胞的功能。MM患者sBCMA表达水平不仅与疾病分期呈正相关[19]，且与浆细胞比例、治疗反应以及生存期具有相关性，另外PD患者的水平高于CR患者，并随着治疗反应的加深而降低[19]–[21]。

接受CAR-T细胞治疗的RRMM患者高表达的sBCMA是否影响CAR-T细胞的疗效？sBCMA可能是与MM患者肿瘤负荷和预后相关的生物标志物[20]–[21]，然而，没有临床证据表明患者sBCMA表达水平会影响BCMA CAR-T细胞治疗的疗效与安全性。

我们的研究中，初步探索了29例RRMM患者CAR-T细胞治疗与其骨髓sBCMA表达的相关性。我们观察到，获得OR的RRMM患者骨髓sBCMA表达，在治疗前高于治疗后，而获得MR+SD患者未观察到该情况；值得注意的是CAR-T细胞治疗前，RRMM患者骨髓sBCMA表达OR组患者低于MR+SD组。RRMM患者骨髓sBCMA表达可否作为预测BCMA CAR-T细胞疗效的指标有待我们进一步观察验证。

结合文献报道，MM患者中sBCMA的半衰期和临床状态的变化比M蛋白提前[22]，sBCMA可快速反映浆细胞负荷变化，使其成为潜在的早期重要监测手段[21]。总结我们的研究结果，MM患者骨髓sBCMA表达或可作为MM患者在诊断、复发和随访期间的监测标志物，及RRMM患者接受BCMA CAR-T细胞治疗的疗效预测标志物，但RRMM患者入组CAR-T细胞治疗前MM细胞的BCMA表达水平与疗效及安全性无关。
